# Direct Measurement of Density for Evaporated Thin Films

**DOI:** 10.1002/smtd.202501438

**Published:** 2025-09-28

**Authors:** Trevor Plint, Halynne R. Lamontagne, Joseph Manion, Benoît H. Lessard

**Affiliations:** ^1^ Department of Chemical and Biological Engineering University of Ottawa 161 Louis Pasteur Ottawa ON K1N 6N5 Canada; ^2^ Department of Chemistry and Biomolecular Sciences University of Ottawa 150 Louis Pasteur Ottawa ON K1N 6N5 Canada; ^3^ School of Electrical Engineering and Computer Science University of Ottawa 800 King Edward Ave Ottawa ON K1N 6N5 Canada

**Keywords:** carbon‐based semiconductors, experimental film density, high vacuum thermogravimetric analysis, organic electronics, thin films

## Abstract

A simple and elegant method is reported for direct measurement of the density of vapor‐deposited thin films using a combination of profilometry, microscopy, and high‐vacuum thermogravimetric analysis (TGA). Density affects fabrication control, optical properties, charge transport, and mechanical properties of thin film devices. Accurate density determination is essential for optimizing device design to ensure film stability, charge balance, and light‐matter interactions. Exact mass of the vapor‐deposited thin films with suitable mass and aspect ratio is determined by high‐vacuum TGA. Combined with precise measurements of thickness and area for each film, the true density of individual films is captured as deposited. This method avoids common universalizing assumptions about the degree of crystallinity in a film. Furthermore, this method does not depend on bulk optical or electrical parameters that may vary as a result of the unique nanoscale properties of thin films. Density values are reported for a range of small molecule semiconductors commonly found in organic electronics such as organic light emitting diodes (OLEDs), organic photovoltaics (OPVs), and organic thin film transistors (OTFTs), and compared to values calculated by other means. It is anticipated that this technique can be used to measure the density of a wide range of vacuum‐stable thin film materials.

## Graphical abstract

We present a simple, direct method to measure the true density of vapor‐deposited thin films using profilometry, microscopy, and high‐vacuum TGA. Unlike conventional approaches, it avoids assumptions about crystallinity or bulk properties. Demonstrated across OLED, OPV, and OTFT materials, this technique provides accurate density values critical for optimizing film stability, charge transport, and light–matter interactions.

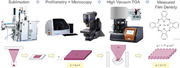

## 1 Introduction

Density is a macro‐scale property that represents the sum of nanoscale molecular attractions and repulsions within a film. These interactions are sensitive to local conditions, for example interfacial templating effects,^[^ ^1^ ^]^ substrate temperature,^[^ ^2^ ^]^ film deposition rate,^[^ ^3^ ^]^ recrystallization effects, and the presence or absence of low‐concentration dopants or contaminants.^[^ ^4^ ^]^ For organic electronic materials, the intermolecular spacing governed by density has direct causal effects on key performance metrics including charge carrier mobility,^[^ ^5^ ^]^ optical properties,^[^ ^6^ ^]^ Förster resonant energy transfer (FRET),^[^ ^7^ ^]^ charge recombination rates,^[^ ^8^ ^]^ and chemical stability.^[^ ^9^ ^]^ Density is also an input to methods for determining material properties, such as mechanical strength, porosity, and refractive index. Incorporating inaccurate density data to these calculations can lead to incorrect assumptions about the properties of thin films. Computer models have been developed that use stochastic deposition simulation to attempt to predict molecular orientation and morphology in silico.^[^ ^10^ ^]^ However, tuning these models to give reliable results depends on either a strong first principles analytical model of film growth and annealing, or else on high quality measured data to calibrate the model. Lacking a universally accepted generalized approach to the former, it is of value to measure the density of materials of interest to the field. It is particularly important to do so in a way that preserves the individual peculiarities of their thin film characteristics.

Density is measured in mass per volume. For a typical thin film that has a surface area on the order of 100 mm^2^ and thicknesses on the order of 100 nm, the mass will be on the order of 10 µg. The area values are easily quantified using modern optical instrumentation. The thickness values require specialized equipment such as contact profilometry or atomic force microscopy (AFM), and are affected by the roughness or unevenness in the film. The mass of these films is the most difficult aspect to measure; 10 µg is at the edge of what can be detected with commonly available balances, and is on a similar order of magnitude to contaminants such as dust particles or contact residues. For this reason, methods of estimating mass have historically been the main bottleneck to obtaining direct measurements of thin film density. Various experimental techniques have been developed over the years to measure the density of materials used in thin film devices. Some examples of these methods are listed in **Table** **1**.

**Table 1:** Various methods used to calculate density of evaporated small molecule thin films.

Method	Description	Example materials	Refs.
Pycnometry	Measures density of bulk powders using a divided gas cell with calibrated volume. Assumes thin film has the same density as bulk powder.	SnPc	[51]
Floatation	Measures density of solid piece of material by displacement of a liquid in which the molecule has negligible solubility. Assumes thin film has same density as bulk material	Perylene	[52]
Optical absorption	Measures optical absorption of thin film on transparent substrates. Beer‐Lambert calibration curve is used to calculate mass of film. Assumes optical absorption spectra of thin films and solutions are equivalent.	Tetracene, Alq_3_, NPB, CBP	[15, 53]
Ellipsometry	Measures changes in refractive index to determine “relative density”. Assumes a standard reference density value to calculate film density, and linear changes in density with respect to morphological composition.	DPAVBi‐doped TBADN (95w/5w), NPB	[9, 54]
X‐Ray diffraction/X‐Ray Reflection	Measures diffraction pattern of X‐Rays through or off of a thin film, bulk solid, or isolated single crystal. Calculates density from inferred atomic packing motif. Assumes density of thin film is equivalent to that of the crystal motif.	TPD, Alq_3_, Cl‐BsubPc, TiO_2_	[55, 56, 57, 58]
Simulation methods	Calculates density from predicted end geometry of atoms or molecules. Uses stochastic methods to simulate film growth. Assumes model output adequately reflects real world deposition conditions.	Molybdenum, NPB, CBP	[59, 60]

Methods like pycnometry that infer density based on data derived from bulk materials like powders or crystals have the advantage of access to pure solid material unconnected to a substrate. However, these approaches have the disadvantage of assuming that the relative ordering of the bulk material is replicated on the substrate after passing through the intrinsically disordered gas or liquid phase during device fabrication. Furthermore, nanoscale structures are often created precisely because they behave differently than their bulk counterparts. Given that the initial conditions at the substrate may vary considerably from those present during the formation of the bulk solid, this assumption introduces significant uncertainty into the determination of film density. Methods that use some form of X‐Ray Diffraction (XRD) are particularly sensitive to extrapolations from the density of single crystals to that of a thin film. Thin films deposited at rates on the order of 1 Å s^−1^ typically show some variable degree of amorphous character.^[^ ^11^ ^]^ This can lead to significant divergence between the measured density of a single crystal and the actual density of a thin film.

Optical methods to measure film density typically make use of the inferred linear correlation between mass concentration, and the magnitude of optical absorbance, usually described by the Beer‐Lambert law. If the optical path length through a film can be determined, then mass density along that path length can be calculated using a calibration curve derived from solution absorbance data. Essentially, this approach treats the thin film as a solid, solvent‐free solution. This method has the advantage of direct measurement of the thin film as deposited, and captures the effects of run‐to‐run variation. However, it depends entirely on the assumption that the optical properties in solid state and solution will be identical. Many organic semiconductor materials are known to exhibit differences between their solution and solid state absorption spectra. These include changes to peak wavelength due to solvent interactions,^[^ ^12^ ^]^ as well as changes to peak magnitude^[^ ^13^ ^]^ due to aggregation effects in the solid state. Furthermore, solid state absorption spectra are known to change depending on the degree of crystallinity, or even between crystal polymorphs of the same material.^[^ ^14^ ^]^ These effects are not captured by solution absorbance spectra, and may result in divergence between the optically implied density and the actual density. Furthermore, some materials of interest to the space exhibit poor solubility, making this method ineffective from the start.^[^ ^15^ ^]^ Therefore, not all materials are suitable candidates for absorbance‐based methods of density determination.

Simulation methods to predict the properties of thin film have shown rapid advances in recent years.^[^ ^16^ ^]^ These methods have both the advantage and drawback of avoiding direct measurement of physical materials. A fully *a‐priori* method capable of accurately predicting the density of thin films would be of great value to the field of device design. However, current approaches struggle to quantitatively account for real‐world effects such as substrate surface energy, low‐concentration contaminants, and variations in deposition rate. Furthermore, simulation methods may make use of empirical data for the purposes of model calibration. It is therefore important that empirical measurements of thin films be made available so that simulation models can be developed that provide the most accurate possible results.

Finally, the degree of crystallinity in a thin film can change over time. Typically, thin films will start at a maximum level of disorder, and may slowly become more crystalline over time as the molecules in the film rearrange toward energy minimizing configurations.^[^ ^17^ ^]^ Depending on the application, this film re‐crystallization may be desirable or undesirable. For devices where charge carrier mobility is paramount, increasing the degree of crystallinity may lead to improved performance.^[^ ^18^ ^]^ However, in devices where aggregation induced quenching is to be avoided, the loss of the initial glassy character of the film may lead to performance loss and even chemical degradation.^[^ ^19^, ^20^ ^]^ Therefore, when precise values of thin film density are called for, it is useful to have a method to capture the density of the film as it exists at the time of measurement.

In this work, we apply thermogravimetric analysis (TGA) under high vacuum to measure the mass of thin films. TGA is a well established technique for observing changes in mass to a material as a function of changes in temperatures.^[^ ^21^ ^]^ In pure materials, the sublimation rate is strongly tied to a specific temperature, making the mass loss associated with a specific material easy to isolate, and often diagnostic of the material. Materials used to make thin films by physical vapor deposition (PVD) are typically vacuum‐stable. By replicating the temperature and pressure conditions of the PVD process, one can re‐evaporate the film, while capturing the exact mass loss over its return to the gas phase. By operating under vacuum, the potential for chemical degradation of the film material is minimized.

Thin films of 50 nm contain too little mass to be accurately measured using our TGA. Since the reorganization energies and steric interactions that govern film growth are localized to the surface during growth, thin film morphology is assumed to be independent of film thickness after the initial monolayers. Thus, the density of a > 1 µm thick film should represent the density of a film of more representative thickness used in organic electronics (e.g. 10 nm – 100 nm). In order to obtain films with enough mass for detection by TGA, we deposit films that are an order of magnitude thicker than conventional devices. By dividing the mass of the film with the measured volume of the film, one can calculate the density of a specific film.

**Figure 1:**
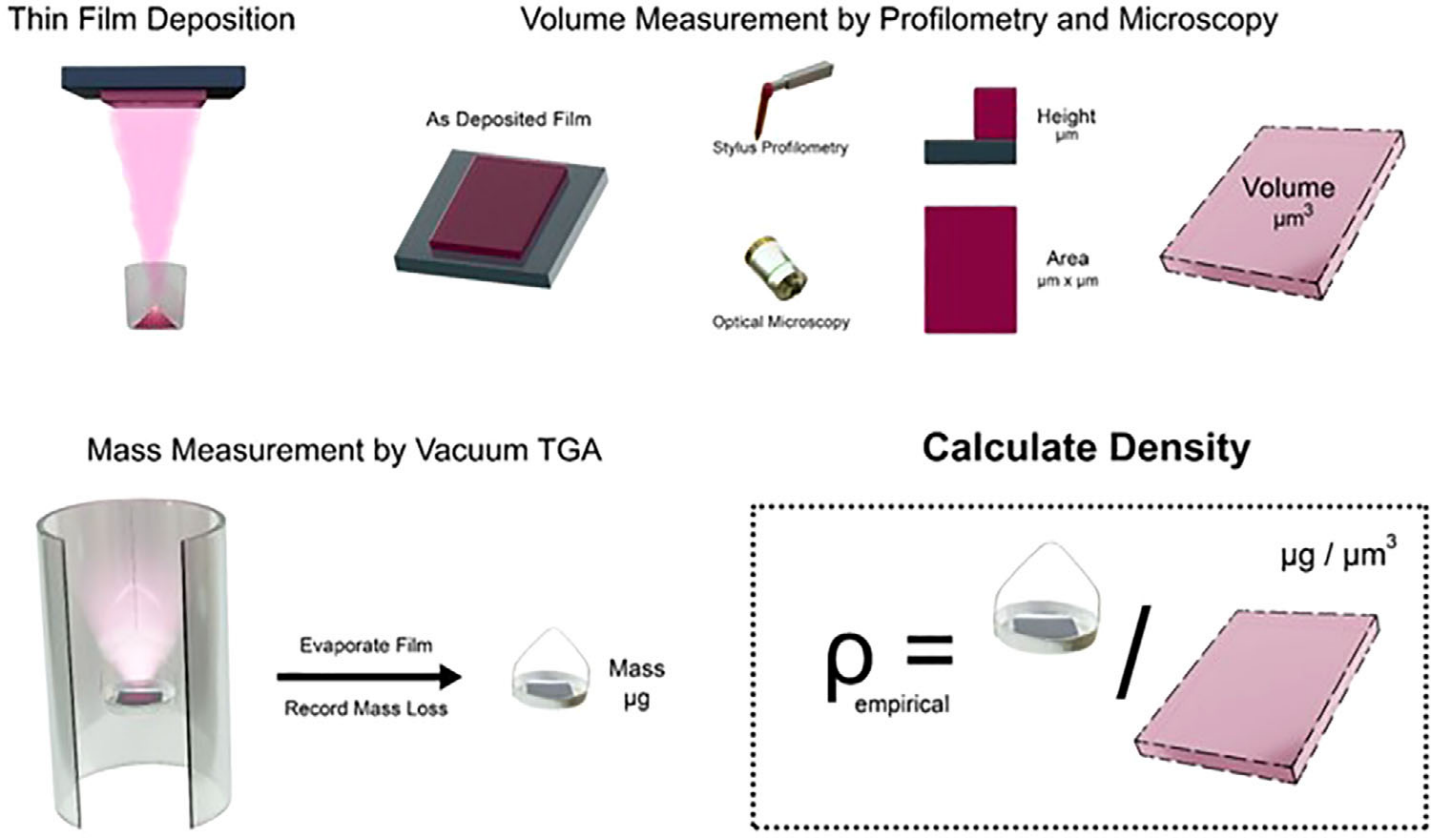
Schematic showing the determination of evaporated thin film density using a combination of profilometry, microscopy, and high vacuum TGA.

The method described in this work has advantages over other methods of film density determination: it makes use of as‐deposited films, accounting for sources of run‐to‐run variation in film morphology; it is unaffected by changes to optical absorption properties that may arise due to thin film formation; it automatically corrects for any variations in crystallinity that may occur during or after deposition; and being a quantitative empirical measurement it does not depend on any a‐priori theory of film organization. A schematic of this process is shown in **Figure** **1**.

**Figure 2:**
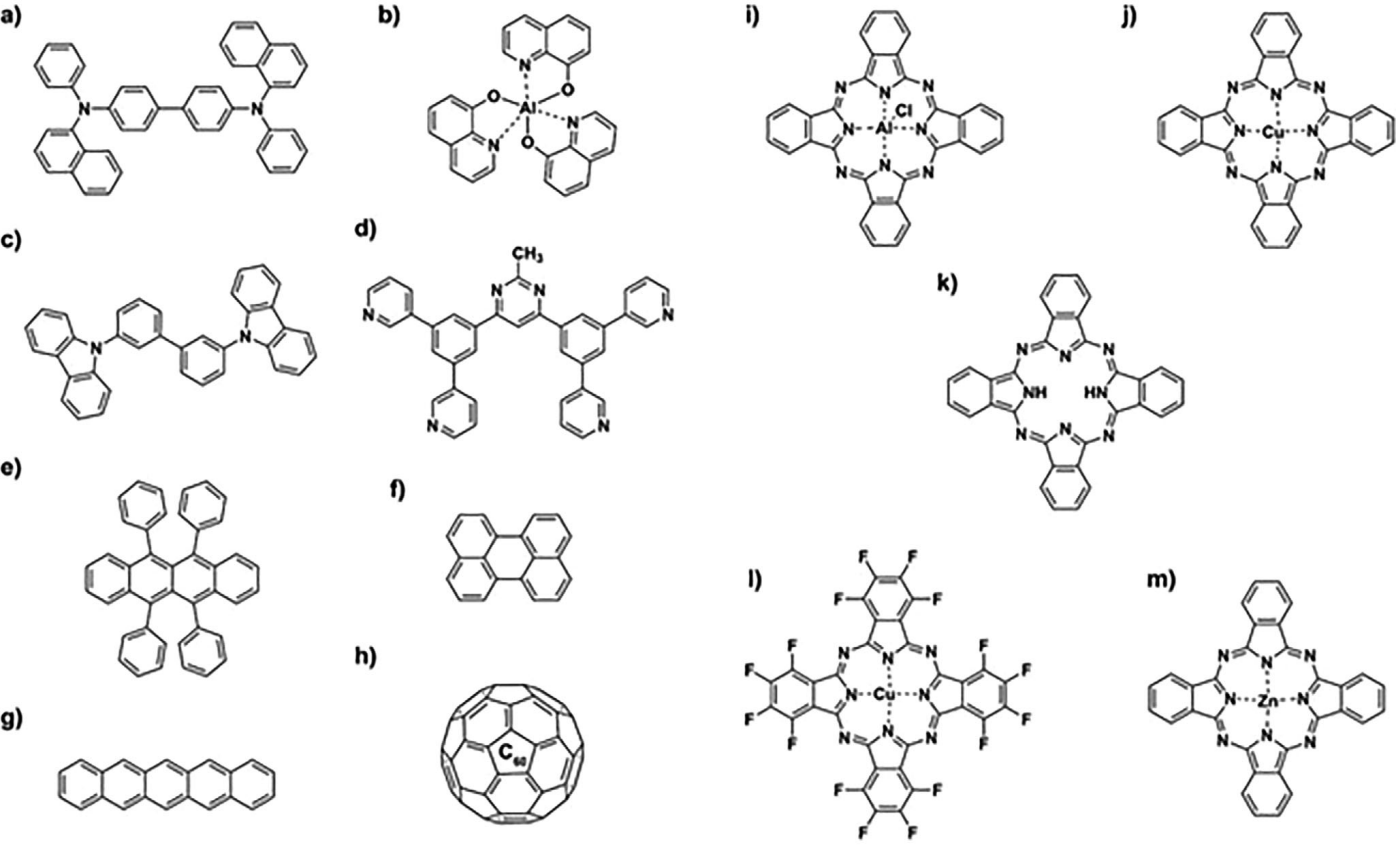
Chemical structures of materials studied in this work. a) NPB b) Alq_3_ c) m‐CBP, d) B3PYMPM, e) Rubrene, f) Perylene, g) Pentacene, h) C_60_, i) Cl‐AlPc, j) CuPc, k) H_2_Pc, l) F_16_‐CuPc, m) ZnPc.

To demonstrate this new method, we have measured the density of a range of commonly used thin film organic semiconductor materials, whose chemical structures are presented in **Figure** **2**. Tris(8‐hydroxyquinoline)aluminum(III) (Alq_3_), 3,3'‐Di(9H‐carbazol‐9‐yl) biphenyl (m‐CBP), 4,6‐Bis(3,5‐di(pyridin‐3‐yl)phenyl)‐2‐methylpyrimidine, 4,6‐Bis(3,5‐di‐3‐pyridinylphenyl)‐2‐methylpyrimidine (B3PYMPM) are used as charge transport layers in OLEDs.^[^ ^22^, ^23^, ^24^ ^]^ Chloro‐aluminum phthalocyanine (Cl‐AlPc), zinc phthalocyanine (ZnPc), copper phthalocyanine (CuPc), copper(II) 1,2,3,4,8,9,10, 11,15,16,17,18,22,23,24,25‐hexadecafluoro‐29H,31H‐phthalocyanine (F_16_‐CuPc), metal‐free phthalocyanine (H_2_Pc), pentacene, perylene, rubrene, and buckminsterfullerene (C_60_) have been studied extensively as charge transport materials in organic thin film transistors (OTFTs),^[^ ^25^, ^26^, ^27^ ^]^ and as photoactive layers in organic photovoltaics (OPVs).^[^ ^28^, ^29^, ^30^, ^31^ ^]^ Films of these materials are typically made via PVD. The electrical, optical, and device properties of these materials have been documented extensively by us^[^ ^26^, ^32^, ^33^ ^]^ and by others. We demonstrate the applicability of this novel approach to a broad range of PVD materials and resulting thin films.

## 2 Results and Discussion

**Figure 3:**
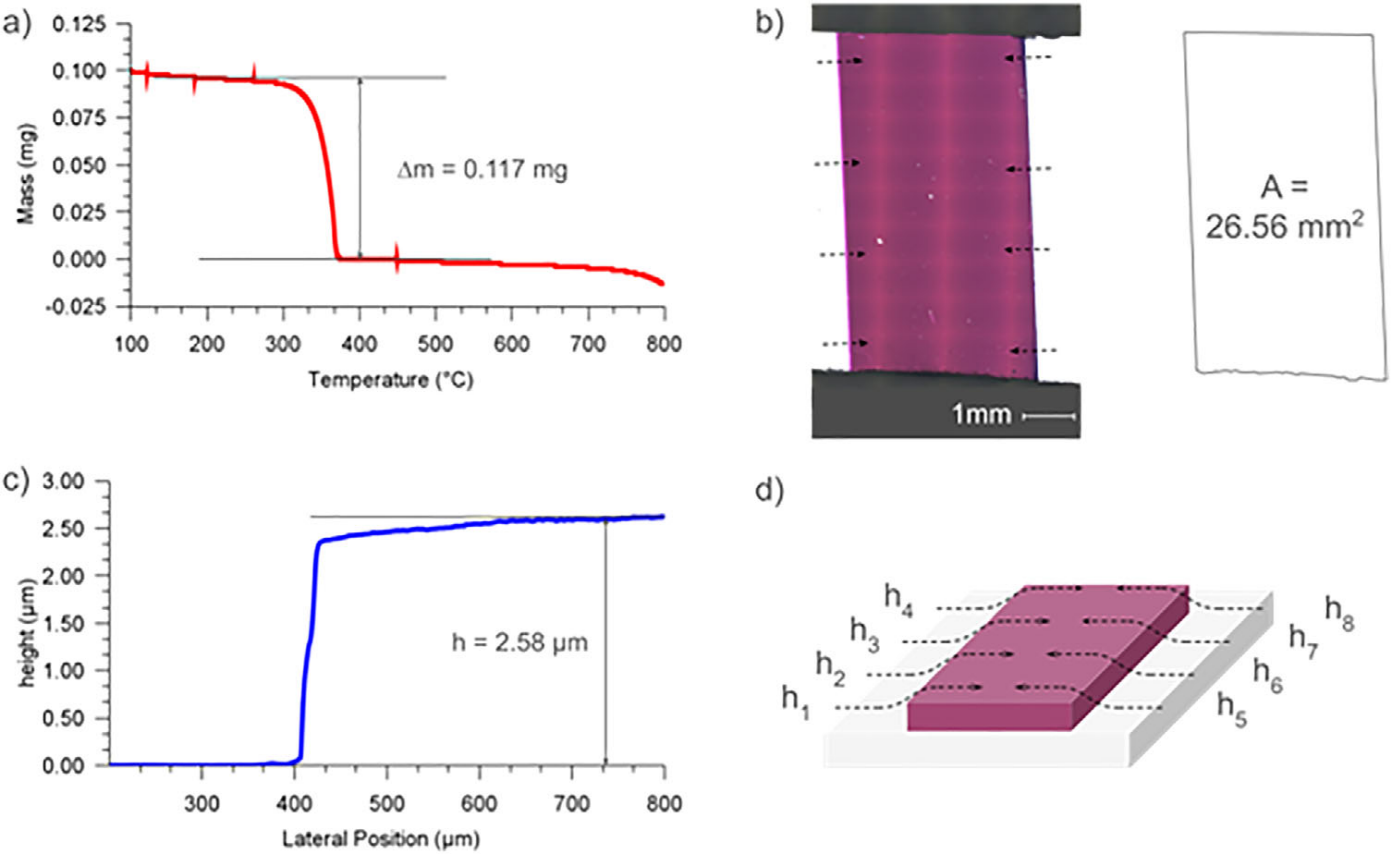
Representative data used to calculate the density of a thin film of CuPc. a) plot of mass as a function of temperature showing mass loss. b) bright field confocal microscope image of thin film. Dashed arrows indicate locations where thickness of the film was measured. Adjacent illustration shows perimeter and calculated surface area for the thin film c) plot of profilometry step height for representative positions on the thin film. d) schematic shows 3d relief of thin film profilometry measurements.

We deposited a series of thin films based on various commonly used organic semiconductors, and measured their densities through our direct high vacuum TGA‐based approach. Since materials used in PVD thin films are typically vacuum‐stable, replicating the temperature and pressure conditions of the deposition process enables the reversal of deposition, while accurately capturing film mass loss. Operating under vacuum further minimizes the risk of chemical degradation. To ensure sufficient mass for TGA detection, thicker films are deposited than what would typically be used in an organic electronic device. The TGA balance has a resolution of <0.1 µg, and the TGA furnace can accommodate films with area on the order of 1 cm^2^. For the Discovery 5500 TGA measurements of good precision can be made for mass changes on the order of 10 µg. For a small molecule semiconductor with density on the order of 1 g cm^−3^, a film thickness of at least 1 µm should be targeted to obtain a precise density value. Silicon wafers were selected as substrates for their high smoothness (<2 Å), and the strongly planar character of their surface. Furthermore, silicon wafers show good temperature stability, reducing the chance of mechanical or chemical changes during heating and cooling. Finally, silicon wafers are relatively easy to cut down to the 8 mm × 10 mm target size. Prior to TGA analysis we calculated the volume of the films by treating films as a regular prism with a definite height and area. The precise area of the films was measured from images captured through optical microscopy, and the overall film thickness measured through stylus profilometry. The product of these two values was taken to be the volume of the film. The mass of the film was measured by re‐evaporation under vacuum in the TGA furnace. The calculated areas, thicknesses, masses, and densities of the materials examined in this study are reported in **Table** **2**. Where available, density values from the literature are also provided for comparison. Representative data for profilometry traces showing thickness, microscope images showing surface area, and TGA traces showing mass loss for CuPc are shown in **Figure** **3**. Detailed plots showing all TGA results are collected in Figure S1 (Supporting Information). All profilometry traces for each film are collected in Figure S2 (Supporting Information). Microscope images of each film are collected in Figure S3 (Supporting Information).

**Table 2:** Measured thickness, surface area, mass and calculated density for thin films of various small molecule organic semiconductors.

Material	Average film Thickness [µm]	Film area [mm^2^]	Film mass[mg]	Density (this work, g cm^−3^)	Density (literature values, if available, g cm^−3^)
NPB	7.91	27.70	0.289	1.32^f)^	^e)^
Alq_3_	9.71	14.39	0.197	1.41^f)^	1.49^[^ ^61^ ^],^ ^a)^, 1.31^[^ ^15^ ^],^ ^b)^, 1.37^[^ ^35^ ^],^ ^a)^, 1.118^[^ ^62^ ^],^ ^c)^
Alq_3_	4.81	29.29	0.193	1.37^g)^	
m‐CBP	2.43	17.65	0.065	1.52^f)^	^e)^
B3PYMPM	2.03	17.95	0.062	1.70^f)^	^e)^
Rubrene	7.49	27.79	0.269	1.29^f)^	1.26^[^ ^39^ ^],^ ^d)^, 1.263^[^ ^39^ ^],^ ^a)^
Perylene	5.66	26.80	0.228	1.50^f)^	1.35^[^ ^52^ ^],^ ^d)^
Pentacene	3.46	27.38	0.135	1.38^f)^	1.32^[^ ^40^ ^],^ ^d)^, 1.365^[^ ^41^ ^],^ ^a)^
C_60_	4.49	26.02	0.206	1.67^f)^	1.65^[^ ^42^ ^],^ ^a)^
C_60_	5.03	24.48	0.206	1.77^g)^	
Cl‐AlPc	3.61	18.60	0.096	1.43^f)^	^e)^
CuPc	2.57	26.56	0.117	1.71^f)^	1.54^[^ ^15^ ^],^ ^b)^, 1.64^[^ ^43^ ^],^ ^a)^
H_2_Pc	7.31	25.16	0.231	1.26^f)^	1.72^[^ ^45^ ^],^ ^a)^
ZnPc	8.34	25.76	0.034	1.58^f)^	1.55^[^ ^44^ ^],^ ^c)^
F_16_‐CuPc	14.68	24.73	0.219	0.60^f)^	^e)^

^a)^ Calculated using X‐ray diffraction data;

^b)^ calculated using optical absorbance spectra;

^c)^ calculated using X‐Ray reflection data;

^d)^ calculated from bulk powder;

^e)^ no thin film density data reported;

^f)^ deposition rates varying between 10 and 100 Å s^−1^;

^g)^ deposition rate 1 Å s^−1^.

The densities of NPB, Alq_3_, m‐CBP, and B3PYMPM were measured to be 1.32 g cm^−3^, 1.41 g cm^−3^, 1.52 g cm^−3^, and 1.70 g cm^−3^, respectively. The measured density values for Alq_3_ are similar to those reported by others.^[^ ^15^, ^34^, ^35^ ^]^ These materials are typically used in OLEDs as charge transport layers, or as host materials.^[^ ^22^, ^23^, ^24^ ^]^ The light outcoupling properties of these layers are critical determinants of the overall efficiency of these devices.^[^ ^36^ ^]^ Outcoupling efficiency is strongly tied to refractive index,^[^ ^36^ ^]^ which is in turn strongly dependent on the density of a material.^[^ ^37^ ^]^ Accurate density information for films of these materials as deposited can help device engineers design more efficient OLED devices. These materials may also be co‐deposited with small amounts of other molecules to obtain doped films with the charge carrier mobility or luminance properties required for the application.^[^ ^38^ ^]^ During co‐deposition, the film thickness and growth rate is measured using a QCM. However, density is one of the input parameters to determine the ratio of materials accumulating on the sensor. Accurate density information for evaporated layers of these materials is a prerequisite to designing and measuring precise doping concentrations, a critical feature of OLED devices and thermoelectric devices.

The densities of rubrene, perylene, pentacene, and C_60_, Cl‐AlPc, CuPc, H_2_Pc, ZnPc, and F_16_‐CuPc were measured to be 1.29 g cm^−3^, 1.50 g cm^−3^, 1.38 g cm^−3^, and 1.77 g cm^−3^, 1.43 g cm^−3^, 1.71 g cm^−3^, 1.26 g cm^−3^, 1.58 g cm^−3^, and 0.60 g cm^−3^, respectively. The values measured for rubrene, pentacene, and C_60_ are remarkably close to values reported elsewhere.^[^ ^39^, ^40^, ^41^, ^42^ ^]^ The measured value for perylene is higher than the 1.35 g cm^−3^ reported by O'Neil et al, however it is unclear which methodology was used to reach this value. The measured values for ZnPc and CuPc are similar to values reported elsewhere.^[^ ^15^, ^43^, ^44^ ^]^ The measured value for H_2_Pc is significantly lower than the value reported by Engel et al.^[^ ^45^ ^]^ However, we note that the values from Engel are derived from measurements of single crystal data, which would likely have significantly different morphology from films deposited at high evaporation rates. We would expect a single crystal to show higher density compared to an amorphous film prepared by vapor deposition. It is worth noting that Xiang et al. attempted to measure the density of H_2_Pc by estimating film mass relative to the absorption spectra in solution. However, they note that the poor solubility of H_2_Pc makes it unsuitable for absorption based density measurement.^[^ ^15^ ^]^ The method presented in this work solves this issue for materials with low solubility, low optical absorbance, or both, since the TGA measurements are not affected by the optical properties of the material. The density value for pentacene reported by Ruiz et al. also indicates the value of measuring directly from thin films where possible. Their efforts are based on crystal density data for the ɑ and β polymorphs of pentacene. However, they note that during deposition of thin films, a layer of ɑ/β character is formed, which complicates the nature of the calculation. They further note the difference in pi‐pi stacking between these two polymorphs, which indicates the importance of film density as a predictor of charge transport behavior. The low density value measured for F_16_‐CuPc is unusual, and suggests that films evaporated at high deposition rates have an especially low packing efficiency. This is corroborated by profilometry measurements, which showed a high degree of surface roughness., Microscope images showed an unusual “shag carpet”‐like surface texture, which appears to be the result of organization into fragile irregularly spaced columnar structures. Profilometry measurements at 1 mg stylus mass loading left pronounced indentations in the surface of the film, indicating a very loosely packed structure with a high degree of porosity. Images of the surface and a representative profilometry trace are collected in Figure S4 (Supporting Information). These results corroborate the low density value measured for F_16_‐CuPc. This unusual morphological effect was not observed for films made from other materials used in this study. This phenomenon illustrates the importance of measuring the true density of as‐deposited film, as it may diverge significantly from single crystal behavior. Phthalocyanine derivatives have been extensively used in experimental OPVs^[^ ^28^, ^29^ ^]^ and OTFTs.^[^ ^25^, ^26^ ^]^ OPVs are dependent on their ability to move charge according to the intrinsic voltage bias inherent to the energy discrepancy between the donor and acceptor regions. Both charge splitting and charge hopping in OPVs is strongly modulated by intermolecular spacing,^[^ ^46^ ^]^ which is in turn a direct function of film density. Although under external bias, OTFTs are subject to similar constraints. Therefore, good empirical data for the density of thin films is useful for designing and predicting the properties of OPVs and OTFTs.

Aspects of the fabrication method used to make the thin films used in this study may limit the applicability of the measured density values. Films with these dimensions are significantly thicker than those found in most organic electronic devices. We believe that the films in this study would exhibit proportionally more bulk character, and less interfacial character. The reported densities for the materials in this work may therefore be different from the densities of films found in organic electronic devices. We anticipate that this discrepancy would be strongest for materials deposited into very thin layers, and in particular for materials known to exhibit strong interfacial templating effects, a category that include phthalocyanines.^[^ ^47^ ^]^

In order to achieve films on the order of 1 µm with reasonable mass utilization efficiency and deposition times, silicon substrates were positioned very close to the evaporative sources. However, due to the close throw distance of our experiment, the deposition rates were significantly higher than those typically used to make thin film devices. Deposition rates varied from sample to sample, but were generally on the order of 100 Å s^−1^. This rate is much higher than the rates ≈ 1 Å s^−1^ that would typically be used to make experimental thin film devices. The deposition rates used in industrial vapor deposition processes are typically not made public, however there are reports of deposition rates up to 100 Å s^−1^ being used to maximize production efficiency.^[^ ^48^ ^]^ In order to examine the effect of elevated deposition rate on measured film density, we fabricated films of Alq_3_ and C_60_ deposited at rates of ≈ 100 and 1 Å s^−1^. The results from these comparative experiments are collected in Table 2. The calculated density values for films of Alq_3_ deposited at 100 and 1 Å s^−1^ were 1.41 and 1.37 g cm^−3^, respectively. The calculated density values for films of C_60_ deposited at 100 and 1 Å s^−1^ were 1.67 and 1.77 g cm^−3^, respectively. The measured density values for these materials show some variation between the fast and slow deposition conditions, but do not follow an obvious trend. In general, one would expect faster deposition rates to correlate with lower density, but the reverse was observed for the C_60_ film. It is unclear what factors contribute to the discrepancy between the Alq_3_ and C_60_ films. In order to examine this effect more closely, AFM was used to measure the surface morphology of C_60_ films deposited at fast and slow deposition rates. These images of these films are collected in Figure S6 (Supporting Information). We observe that measurements of the film deposited at 1 A s^−1^ showed larger grain sizes relative to the film deposited at 100 A s^−1^, a result which is in line with measurements and film growth theory reported elsewhere.^[^ ^49^, ^50^ ^]^ In general, this suggests that thick films deposited at fast and slow deposition rates may exhibit different morphologies. Therefore, for certain materials, accurate density values may require film fabrication at low deposition rates analogous to those used in the device deposition.

Film thicknesses of at least 1 µm were targeted to ensure sufficient mass was present on the 4 mm x 8 mm target area for detection during re‐evaporation. Due to the close throw distance between the substrate and the crucible, step edge thickness was expected to vary smoothly as a function of distance from an angle relative to the evaporative source. The variance in film thickness was calculated by dividing the standard deviation for eight measurements by the mean thickness value. For the thickness values presented in Table 2, film variance ranged between 3.3% and 8.2%. Given the smooth continuous nature of the variation in thickness, it is reasonable to approximate the volume of the thin film from the geometry of a right‐angle prism with height equal to the average step height, and face area equal to the surface area of the film. The mass loss signals for individual films were well resolved during re‐evaporation by high vacuum TGA. The sublimation temperatures of the thin films closely matched the sublimation temperatures of the raw material, removing any ambiguity in the identity of the mass signal. The mass of individual films was calculated by subtracting the balance mass before and after the main mass loss signal. To determine whether any material remained on the substrates post‐TGA, two methods were used. The first was visual inspection with a UV lamp (λ_max_ 365 nm) before and after TGA. Before TGA, films showed mild to strong fluorescence relative to bare silicon substrates. After TGA, substrates showed no visible fluorescence, and were visually identical to pristine Si substrates, indicating a clean re‐evaporation of the film. To further confirm complete re‐evaporation of the film, Raman spectroscopy was used to compare substrates after re‐evaporation to pristine Si substrates. These results were compared to spectra of bulk Alq_3_ powder to check for residual Alq_3_ signal. Within the sensitivity of the Raman detector, no traces of Alq_3_ were observable on the substrate, confirming complete re‐evaporation of the Alq_3_ film. The results from this experiment are collected in Figure S5 (Supporting Information). Re‐evaporation of thin films was carried out at pressures ≈ 2.0E‐5 Torr, which is within the typical sublimation range of the materials suggesting the evaporation process would be similar to the deposition process during thin film formation. Further work may enable higher vacuum or higher temperatures to re‐evaporate materials with lower vapor pressures, enabling measurement of the density of as‐deposited films of metals or other composites.

Overall, these values represent our best effort to describe the bulk density of a range of important organic semiconductor materials in vapor deposited thin films. Differences in film thickness and deposition rate resulting from the practicalities of the experimental apparatus may affect the applicability of measurements to research devices. Further work is needed to refine the technique to measure the density of thinner films. Additional studies may shed further light on the effect of deposition rate on the measured density values for different materials. Reporting more accurate density values would improve the ability of researchers to make calculations and predictions of the behavior of thin films found in working organic electronic devices. That said, this work represents a precise and quantitative measure of the mass density for as‐deposited films of organic semiconductors. It resolves a longstanding reliance on data measured from single crystals, and captures the distinctive effects of film morphology on overall film density. We believe this technique represents a breakthrough for the design, simulation, and quantification of thin film devices.

## 3 Conclusion

In this work we have demonstrated a new method for direct measurement of the density of thin films via a combination of profilometry, microscopy, and high vacuum TGA. Density values for thin films are sensitive to environmental factors such a deposition rate, substrate temperature, material purity, and the presence of other materials such as dopants. Density affects important performance parameters for devices incorporating thin films, including charge transport characteristics, doping concentrations, and refractive index. Measurement of thin film density has been attempted through indirect means, including ellipsometry, optical absorption methods, and estimates based on the unit cell density of single crystals. This work represents the first example of direct measurement of density of as‐deposited thin films. We take into account the discrepancies in the film fabrication methods used for this study, and on balance we believe this method will prove useful to researchers working to optimize the design of thin film systems. Though our study focused on vacuum deposited organic materials, the fundamental density calculation relies on reliable measurement of area, thickness, and mass for materials that can be examined through TGA. In future this could be extended to include volatile metals such as calcium, lead, and magnesium, as well as to less volatile materials through upgrades to the furnace and vacuum capabilities of this system. We further believe that good quality measurements of the density of thin films will enable further discoveries by researchers who seek to simulate the properties of thin film materials in silico.

We also propose that comparing thin film densities with corresponding bulk values can yield insight into key film characteristics, including porosity, packing efficiency, molecular orientation, and structural uniformity. These parameters are critically important for nanostructured materials used in catalysis, sensing, energy storage, optics, and flexible electronics, where film microstructure directly influences functional performance. By enabling direct, reliable density measurements with minimal material requirements, this method offers a versatile tool for experimental optimization, process monitoring, and in silico model calibration. It thus represents a significant step toward a standardized, broadly applicable framework for the quantitative characterization of thin films across materials science, chemistry, and applied physics.

## 4 Experimental Section

### Materials

NPB (>99.8%), Alq_3_ (>99.5%), m‐CBP (>99.5%), and B3PYMPM (>99%), were purchased from Luminescence Technology Corporation, and used without further purification. Rubrene (>95%), perylene (>95%), and pentacene (>98%) were purchased from Oakwood Products Inc, and purified by train sublimation prior to use. C_60_ (>99.5%) was purchased from SES Research and purified by train sublimation prior to use. Cl‐AlPc (>95%), CuPc (>95%), and H_2_Pc (>99%) were purchased from Alfa Aesar and purified by train sublimation prior to use. ZnPc (>95%) and F_16_‐CuPc (>98%) were purchased from TCI Chemicals and purified by train sublimation prior to use. Pre‐diced silicon wafers were purchased from NanoFab, University of Alberta, and cut to ∼8 mm x 10 mm squares using a diamond scribe. These dimensions were targeted to fit the TGA weighing pans, which had a basket diameter of 8 mm. These smaller chips were attached to a stainless steel shadow mask with 4 mm linear aperture using small pieces of kapton tape prior to film sublimation.

### Film Evaporation

Thin films were made using an EvoVac thermal evaporator (Angstrom Engineering), controlled by the Aeres recipe and automation software package. To make thin films, a custom substrate holder was installed close to the evaporation source. The throw distance from the lip of the crucible to the substrate was approximately 60 mm. This throw distance was necessary to obtain films of thickness of at least 1 µm using conventional crucible mass loadings. In principle, it is possible to deposit films of 1 µm or more at longer throw distances; in practice depositions at longer distances either yielded too little accumulated mass, or required a greater crucible capacity than was practical for research evaporator systems. For each deposition, ≈100 mg of semiconductor material was loaded into a 1 cc aluminum oxide crucible (C1, R.D. Mathis), which was placed in a resistive heater. The target substrate was then attached to the substrate holder and the chamber sealed. The chamber was evacuated to ≈2.0E‐7 Torr, and the semiconductor material was evaporated at a constant rate controlled by a quartz crystal microbalance (QCM).

### Film Dimension Measurement

After deposition, film thickness was measured by stylus profilometry (DekTakXT, Bruker Vision64 v.5.51, update 6). The measurement arm of the DekTakXT has a vertical resolution of 1 Å, and a rated step height repeatability of <5 Å. For each film a total of eight thickness measurements were taken, with four evenly spaced step measurements taken on each side. Profilometry traces were distributed as widely and evenly as possible to account for variation in thickness across the horizontal plane. Individual profilometry traces were measured from bare areas of the substrate up onto the surface of the film, with a probe tip mass loading of 3 mg. Edge effects at the perimeter of thin films can lead to localized morphology effects, but their spatial extent is small relative to the overall film dimensions. The profilometry scan lengths were set to 1–2 mm in length depending on substrate geometry. Since the films measured were around 4 mm × 8 mm, these measurements yielded step heights that were representative of the overall thickness of the film. After profilometry, images showing the precise geometry of films were captured using a confocal microscope (Olympus DSX10‐UZH, PRECiV DSX v.2.1.1). The surface area of the films was calculated from focussed bright field images using a 20x magnification lens unit. Images were saved with embedded scale bars calculated by the PRECiV software. The superimposed scale bars were used to determine the unit area of each image in pixels per mm^−2^, and image software was used to measure the number of pixels within the boundaries of the film. From this we were able to calculate the precise surface area of the film.

### Atomic Force Microscopy (AFM)

The surface characteristics of C_60_ films were assessed using an atomic force microscope (Bruker Dimension FastScan using Nanoscope Analysis Version 3.00.1). Measurements were taken of a 5 µm x 5 µm region using ScanAsyst Air probes in PeakForce Tapping “ScanAsyst” Mode.

### Raman Spectroscopy

Raman spectra of a bare silicon chip before and after re‐evaporation were captured on a Renishaw inVia Qontor confocal microscope using the Renishaw WiRE 5.5 software. A scan of bulk Alq_3_ powder was performed to identify its characteristic Raman peaks and confirm the complete removal of all material from the silicon surface after TGA analysis. For all scans, a 500 mW, 785 nm laser was used at 10% power and 20x magnification, where 10 acquisitions of 1 s each were collected and summed. On the post‐TGA chip, five locations across the surface of the chip were measured.

### Thermogravimetric Analysis (TGA)

The mass of films was determined using a custom high‐vacuum TGA (TA/Waters Discovery 5500, TRIOS v5.5.1). The weighing resolution of the TGA balance arm was rated as <0.1 µg, and weighing precision was rated as ±0.01%. Silicon chips with a thin film on one side were placed in torch‐cleaned 100 µL platinum pans, and loaded into the TGA furnace. The TGA furnace was carefully evacuated using a turbomolecular pump (Pfeiffer HiPace 80) backed by a rough pump (Edwards RV8) to a base pressure of ≈ 2.0E‐5 Torr as measured by ion gauge (Kurt J Lesker, Model 354C). The chips were preheated to 70 °C for 5 min to bake out any adsorbed moisture. The films were then heated at 10 °C min^−1^ to a maximum temperature of 700 °C, and the resulting mass loss was recorded. Film density was calculated by multiplying the average thickness of the film by the calculated area of the film to obtain the film volume, and dividing the mass of each film by the volume of the film.

## Conflict of Interest

The authors declare no conflict of interest.

## Supporting information



Supporting Information

## Data Availability

The data that support the findings of this study are available from the corresponding author upon reasonable request.
