# Supplementary material for: Direct Measurement of Density for Evaporated Thin Films
Source: Small Methods. 2025 Sep 28;9(11):e01438. doi: 10.1002/smtd.202501438 (PMC12641363; doi:10.1002/smtd.202501438)

**Direct Measurement of Density for Evaporated Thin Films**

Trevor Plint^1^, Halynne R. Lamontagne^1,2^, Joseph Manion^1^, and Benoît H. Lessard^1,3*^

*^1^Department of Chemical and Biological Engineering, University of Ottawa, 161 Louis Pasteur, Ottawa, ON, Canada, K1N 6N5*

*^2^Department of Chemistry and Biomolecular Sciences, University of Ottawa, 150 Louis Pasteur, Ottawa, ON, Canada, K1N 6N5*

*^3^School of Electrical Engineering and Computer Science, University of Ottawa, 800 King Edward Ave, Ottawa, ON, Canada, K1N 6N5*

*Corresponding Authors: [Benoit.Lessard@uottawa.ca](mailto:Benoit.Lessard@uottawa.ca)

**Electronic Supplementary Information**

Figure S1. TGA traces for a) NPB b) Alq_3_ c) m-CBP, d) B3PYMPM, e) Rubrene, f) Perylene, g) Pentacene, h) C_60_, i) Cl-AlPc, j) CuPc, k) H_2_Pc, l) F_16_-CuPc, m) ZnPc


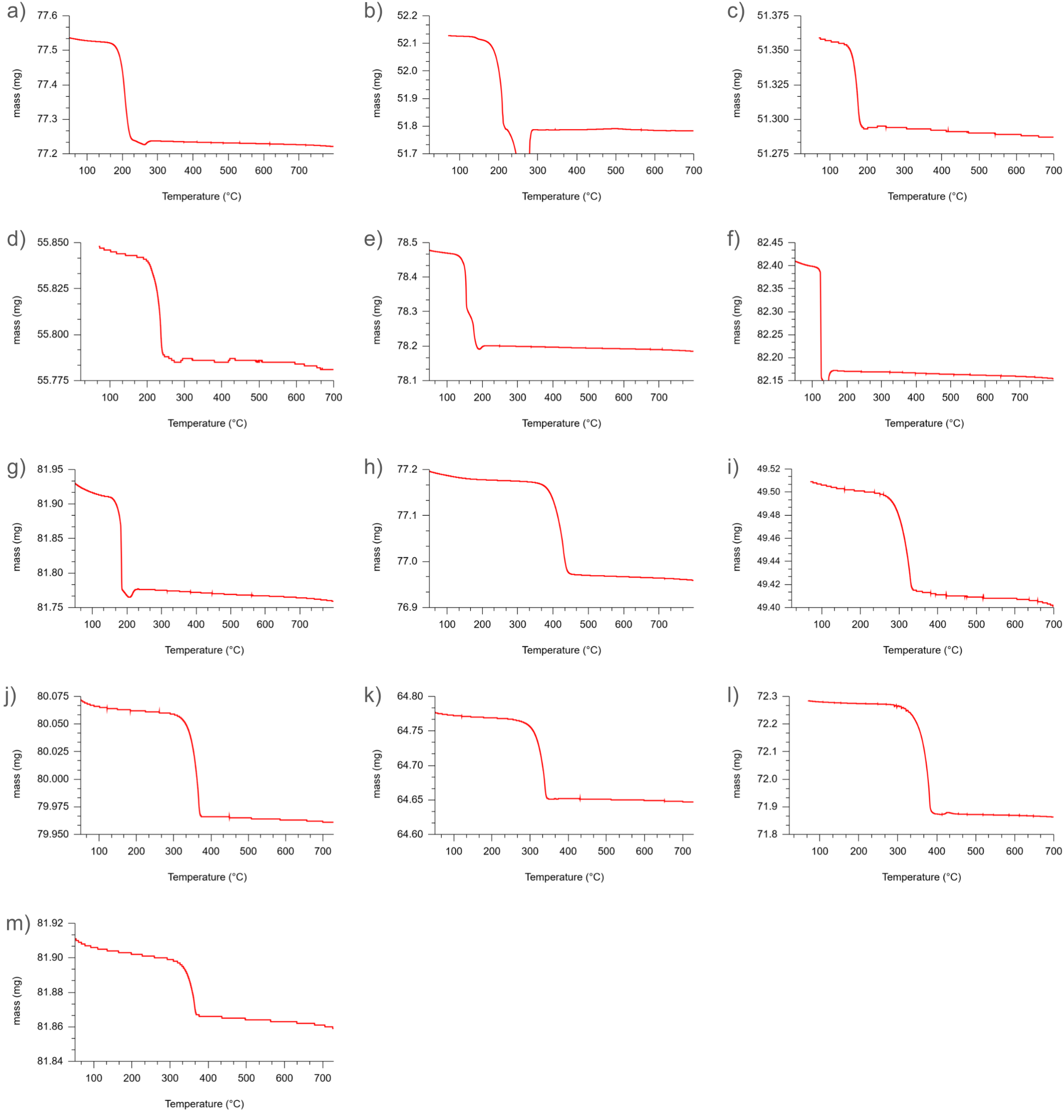


Figure S2. Profilometry traces for a) NPB b) Alq_3_ c) m-CBP, d) B3PYMPM, e) Rubrene, f) Perylene, g) Pentacene, h) C_60_, i) Cl-AlPc, j) CuPc, k) H_2_Pc, l) F_16_-CuPc, m) ZnPc. Films of each material were measured at eight locations distributed as widely as possible across the film surface; each plot shows all eight traces.


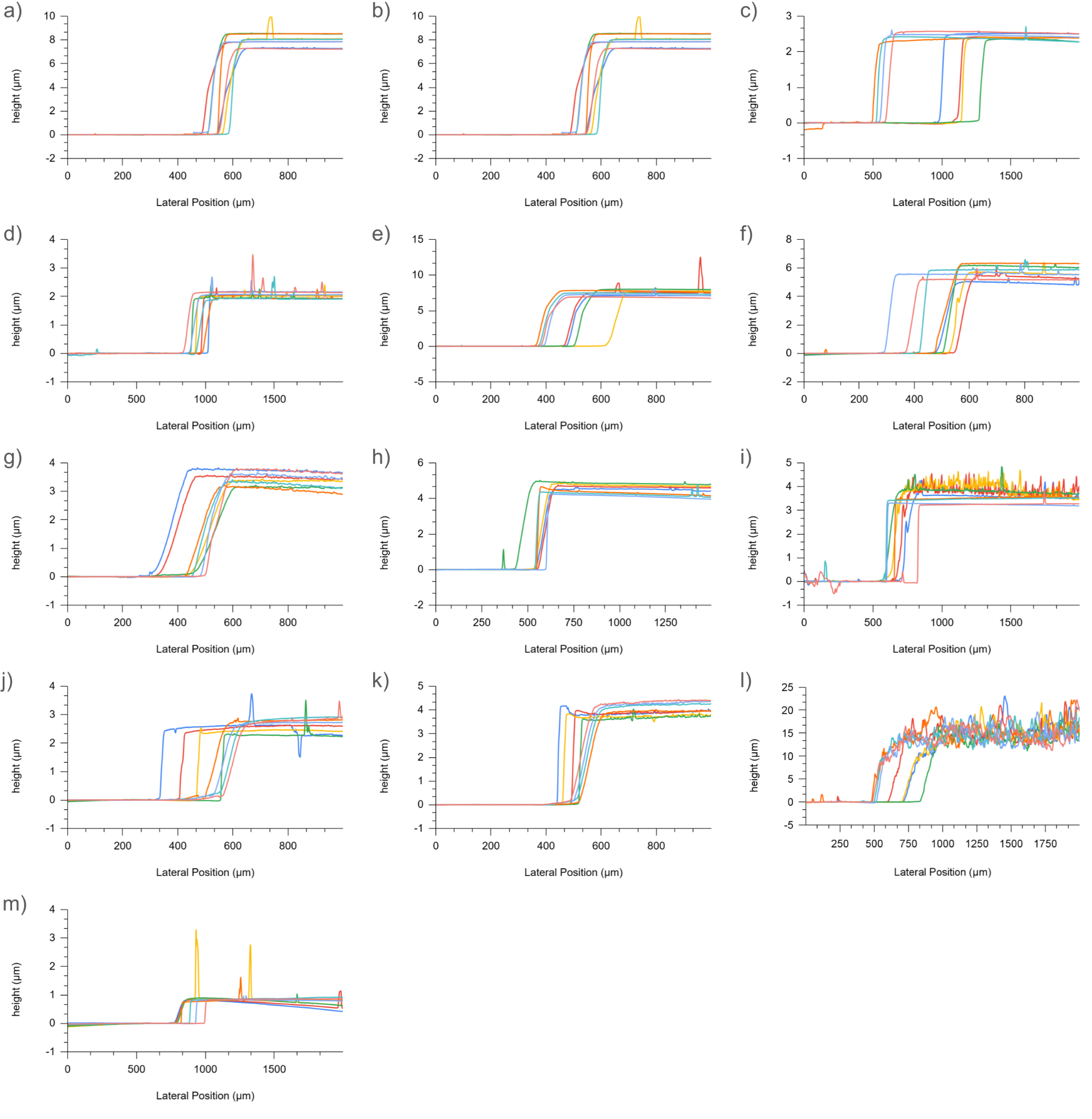


Figure S3. Microscope images for a) NPB b) Alq_3_ c) m-CBP, d) B3PYMPM, e) Rubrene, f) Perylene, g) Pentacene, h) C_60_, i) Cl-AlPc, j) CuPc, k) H_2_Pc, l) F_16_-CuPc, m) ZnPc. Scale bars for each image are adjusted to 1000µm

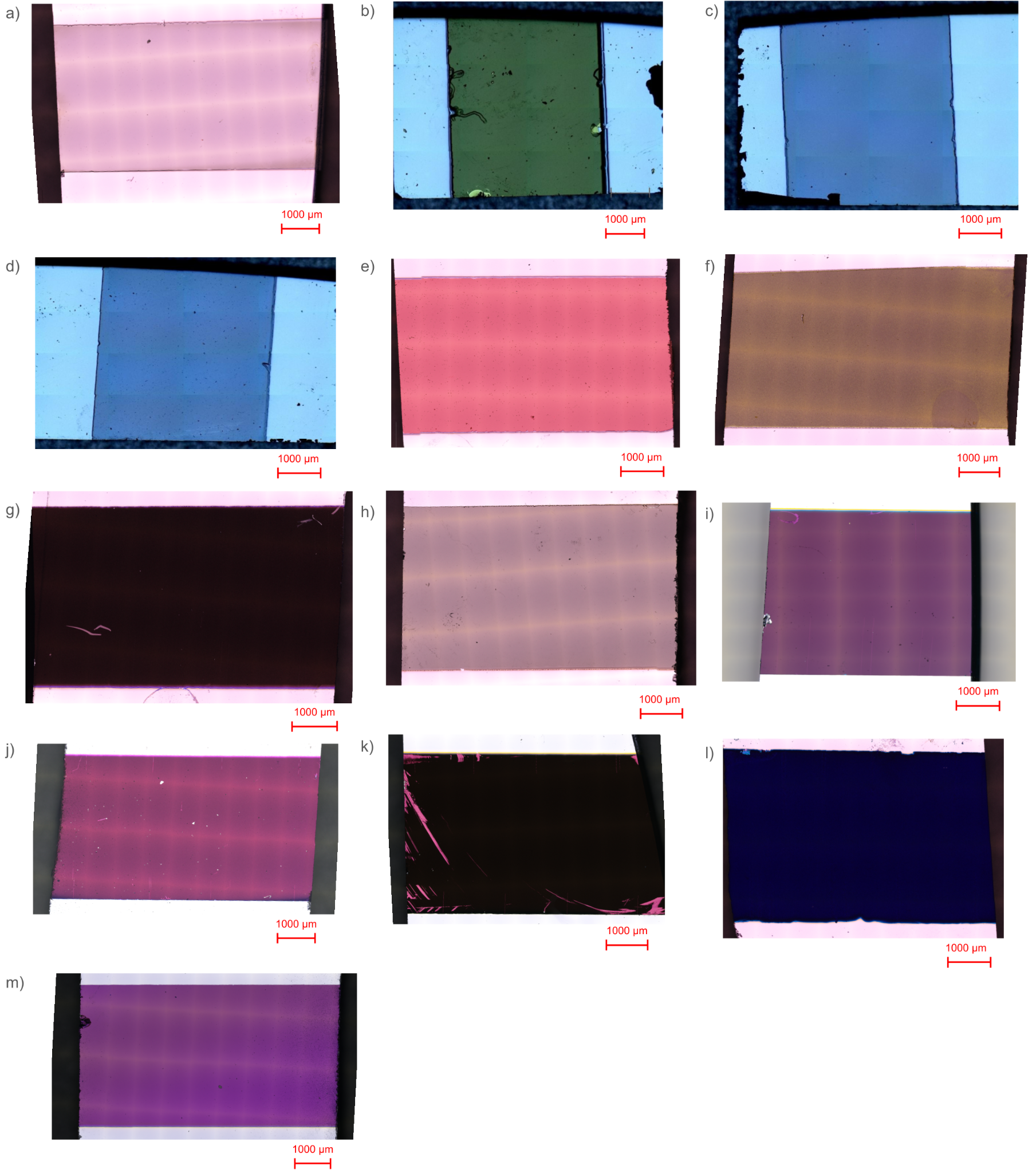


Figure S4. a) Representative profilometry trace for F_16_-CuPc thin film showing significant surface roughness, and b) dark field confocal microscope image of F_16_-CuPc thin film showing shaggy, carpet-like surface made of individual columnar structures. Red arrow indicates where the stylus of the profilometer has dragged through the surface layer, leaving an indentation.


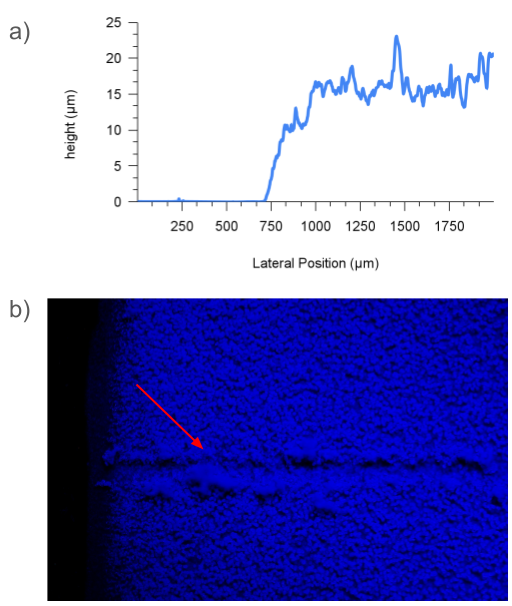


Figure S5 a) Plot of Raman spectra measured for a bare silicon wafer before Alq_3_ film deposition (blue line), and after re-evaporation of Alq_3_ film (red lines) b) microscope image of silicon wafer showing locations of raman scans (red “x” marks), and former area of Alq_3_ film (green dashed line) c) Raman spectra of bulk Alq_3_ powder.


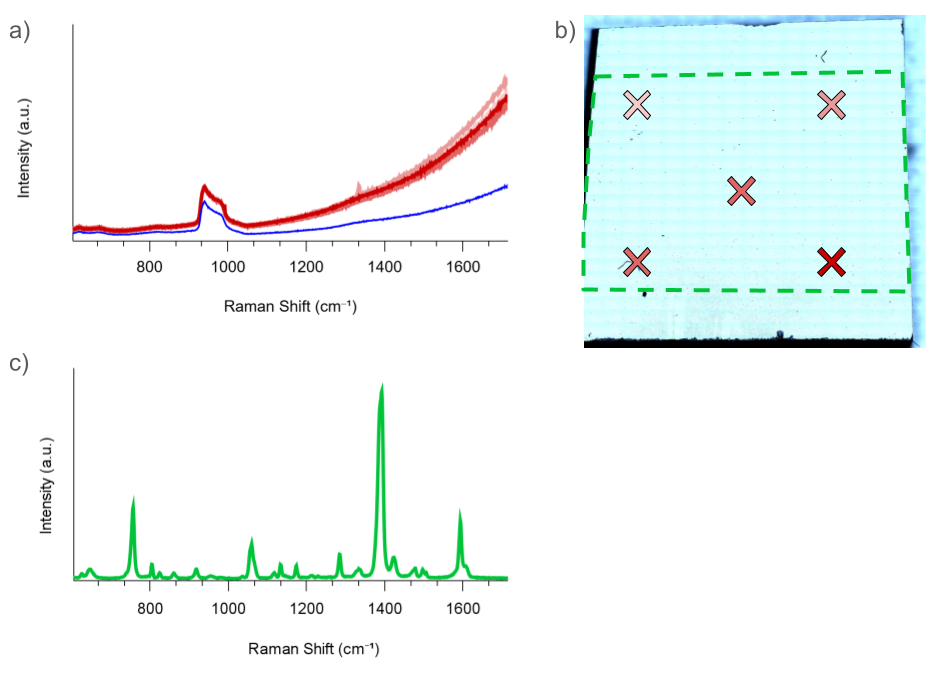


Figure S6. Representative AFM images of C_60_ thin films deposited at a) 1 A s^-1^ and b) 100 A s^-1^.


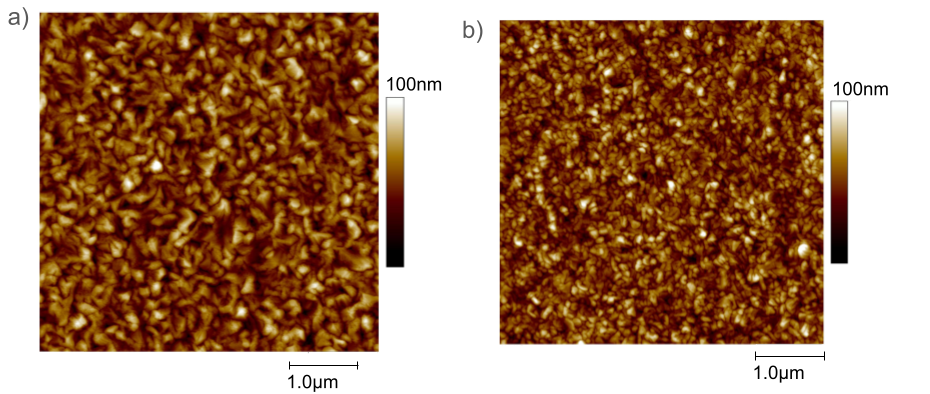

Supplement: Supplementary file 1 — Supporting Information [file SMTD-9-e01438-s001.docx]
